# Diabetic Peripheral Arterial Disease Versus Thromboangiitis Obliterans: A Multidimensional Comparison of Clinical Phenotype, Biomarkers, and Outcomes

**DOI:** 10.3390/diagnostics16040560

**Published:** 2026-02-13

**Authors:** Murat Yücel, Hakan Çomaklı, Muhammet Fethi Sağlam, Kemal Eşref Erdoğan, Nur Gizem Elipek, Ömer Abdullah Yavuz, Emrah Uğuz

**Affiliations:** 1 Department of Cardiovascular Surgery, Ankara Bilkent City Hospital, Ankara 06800, Türkiyegizelipek@gmail.com (N.G.E.);; 2 Department of Cardiovascular Surgery, Ankara Yıldırım Beyazıt University Faculty of Medicine, Ankara 06010, Türkiye

**Keywords:** thromboangiitis obliterans, diabetic peripheral arterial disease, inflammation, biomarkers, diabetic foot, foot ulcer, amputation

## Abstract

**Objective**: This study aimed to compare thromboangiitis obliterans (TAO) and diabetic peripheral vascular disease (DPVD), the two major causes of distal limb ischemia, within a single analytical framework. The comparison was not limited to practical biomarkers that could support differential diagnosis but was based on multidimensional parameters that determine the clinical spectrum and prognosis. The two cohorts were systematically evaluated in terms of demographics and comorbidity burden, clinical presentation and limb involvement pattern, ulcer prevalence and localization, real-life treatment strategies (medical, endovascular, and surgical), and hard clinical endpoints (major/minor amputation, hospitalization, and all-cause mortality). DPVD was phenotyped according to the lesion level as isolated distal, isolated proximal, or multilevel. Within this framework, the isolated distal diabetic peripheral vascular disease (d-DPVD) subgroup was analyzed to determine how it differs from TAO in terms of clinical course, treatment patterns, and outcomes, despite the distal anatomical similarity. **Methods:** In this single-center retrospective cohort study, conducted between June 2019 and June 2025, 120 non-diabetic patients who met the angiographic TAO criteria were compared with 395 patients with DPVD with infrapopliteal/pedal atherosclerotic involvement. Clinical characteristics, ulcer topography, treatment strategies, and outcomes were recorded. The discriminatory value of the blood count and lipid-based inflammatory/atherogenic indices were evaluated using logistic regression and receiver operating characteristic (ROC) curve analyses. Additionally, a separate subgroup analysis was performed for the d-DPVD subgroup, which was considered the closest to the TAO phenotype in this study design. **Results**: Patients with DPVD were significantly older than those with TAO (61.1 ± 12.1 vs. 39.7 ± 7.9 years; *p* < 0.001), and male predominance was more pronounced in the TAO group (94.2% vs. 84.8%). Compared with TAO, DPVD was associated with a higher cardiometabolic comorbidity burden and increased inflammatory and atherogenic indices. Although the overall ulcer prevalence was comparable, DPVD more frequently presented with plantar or proximal ulcers confined to a single extremity, whereas TAO was characterized by bilateral or multi-extremity involvement and distal acral ulceration. Antiplatelet and statin therapy, revascularization, and rates of major amputation, all-cause mortality, and hospitalization were higher in patients with DPVD (all *p* < 0.05). On multivariate analysis, age, cumulative smoking exposure, SIRI, and CRI-I independently distinguished DPVD from TAO (all *p* < 0.05). In the isolated distal DPVD subgroup, despite similar distal anatomy, inflammatory/atherogenic burden, and overall clinical risk remained adverse. **Conclusions:** TAO and DPVD are two distinct phenotypes with different pathobiologies and prognoses, despite similar distal ischemia presentations. Simple inflammatory and atherogenic composite indices, evaluated in conjunction with clinical/ulcer patterns, may support the differential diagnosis and risk stratification of patients with peripheral arterial disease (PAD). However, prospective multicenter validation of these findings is required to confirm the results.

## 1. Introduction

Peripheral vascular disease is a global health problem affecting millions of individuals worldwide, causing morbidity, mortality, and significant healthcare expenditures. Diabetic peripheral vascular disease (DPVD) and thromboangiitis obliterans (TAO), also known as Buerger’s disease, are two fundamental entities characterized by distal limb ischemia, ulceration, and tissue loss. Despite their similar clinical presentations, they are distinctly different in terms of pathobiology [1,2].

DPVD is a chronic systemic disease characterized by atherosclerotic involvement of medium and large vessels, developing on a background of chronic hyperglycemia, endothelial dysfunction, macro- and microangiopathy, neuropathy, and atherogenic dyslipidemia, which strongly predispose patients to critical limb ischemia (CLTI) [3]. In contrast, TAO represents a non-atherosclerotic, segmental inflammatory/thrombotic vasculitis pattern affecting small- and medium-sized vessels, mostly seen in young, heavy smokers [4,5]. Despite being clinically recognized for over a century, it remains a mysterious condition with incompletely understood etiopathogenesis involving complex interactions between tobacco exposure, genetic predisposition, immune dysregulation, and endothelial damage [6].

Recent epidemiological studies have shown that vascular involvement patterns in patients with diabetes are markedly heterogeneous; isolated distal, proximal, and multilevel arterial disease subgroups have different effects on prognosis and revascularization strategies [7]. However, this has not been sufficiently investigated in the current literature. Similarly, in clinical practice, distinguishing between TAO and isolated distal diabetic peripheral vascular disease (d-DPVD) in cases presenting with distal tissue loss and critical ischemia poses a significant diagnostic challenge because of the heterogeneous diagnostic criteria used for TAO and the lack of an agreed-upon “gold standard” diagnostic method [8]. Furthermore, isolated distal and multilevel involvement of the a, which is common in the DPVD population, becomes a critical confounder in comparisons with PAD in terms of the anatomical level and systemic atherosclerotic burden. Current evidence suggests that systemic inflammation and atherogenic dyslipidemia in DPVD may be associated with amputation and mortality [9,10], whereas in TAO, inflammatory markers may be related to the disease activity. However, it remains unclear whether these two conditions represent different ends of the same inflammatory-atherogenic spectrum or biologically distinct entities, particularly in the presence of distal bed involvement. The literature on DPVD and TAO is limited in terms of demographic characteristics, comorbidity burden, clinical phenotype, ulcer prevalence and localization, extremity involvement pattern, real-world treatment strategies (medical–endovascular–surgical), and hard endpoints such as major/minor amputation, hospitalization, and mortality within the same methodological framework are extremely limited [11,12].

Furthermore, the comparative value of systemic inflammatory indices (NLR, SII, SIRI, AISI, and others) and atherogenic ratios (AIP and Castelli risk indices) derived from routine hemogram and lipid profiles in distinguishing these two phenotypes and predicting clinical outcomes remains uncertain [8,13]. In this study, we hypothesized that despite similar distal ischemia, DPVD and TAO will be distinctly differentiated in terms of demographic characteristics, lesion pattern, clinical outcomes, and systemic inflammatory/atherogenic indices. Furthermore, these indices will provide useful biomarkers, particularly for the differential diagnosis and risk classification of d-DPVD and TAO.

The rationale for this study was to evaluate TAO and DPVD not only in terms of inflammatory and atherogenic indices but also in terms of demographic characteristics, comorbidities, clinical presentation, ulcer frequency and localization, extremity involvement pattern, medical/endovascular/surgical treatments applied, and short-term clinical outcomes, thereby establishing a more refined etiology-based risk classification and differential diagnosis framework. In this context, the original aspect of the study is phenotyping DPVD according to anatomical distribution, specifically comparing the “isolated distal” diabetic disease subgroup with TAO, and evaluating the potential discriminatory value of composite inflammatory and atherogenic indices derived from routine laboratory data in distinguishing TAO from DPVD and their relationship with clinical outcomes such as amputation/mortality.

## 2. Materials and Methods

### 2.1. Study Design and Ethical Disclosures

This was a single-center, retrospective, observational cohort study. The study protocol was established in writing prior to the data collection process, and the study was reported in accordance with the STROBE (Strengthening the Reporting of Observational Studies in Epidemiology) guidelines, which are internationally accepted standards for observational studies [14]. The study was conducted at the Ankara Bilkent City Hospital Cardiovascular Surgery Clinic between June 2019 and June 2025. The study protocol was approved by the Institutional Ethics Committee (Approval No. TABED 1-25-1858; 19 November 2025). The study was conducted in accordance with the principles of the Declaration of Helsinki and the Good Clinical Practice guidelines. Patient consent was not required because of the retrospective design; however, patient data confidentiality and anonymity were maintained throughout the study.

### 2.2. Study Population, Patient Selection, and Definitions

**Figure 1:**
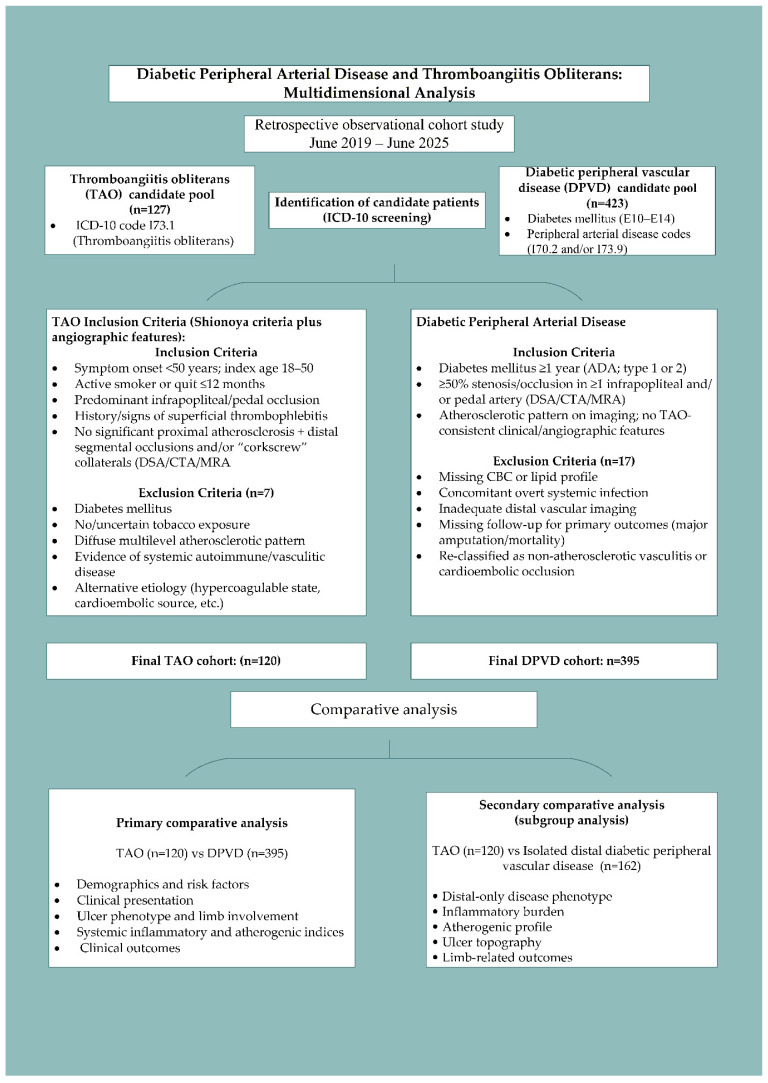
Study flow diagram illustrating patient identification, eligibility criteria, cohort allocation, and comparative analyses between thromboangiitis obliterans (TAO), diabetic peripheral vascular disease (DPVD), and isolated distal DPVD subgroups. Abbreviations: ICD, International Classification of Diseases; CBC, Complete Blood Count.

All patients who visited the Cardiovascular Surgery Outpatient Clinic during the study period were screened using ICD-10 diagnosis codes via the Hospital Information Management System (HBYS), and potentially eligible cases were identified for inclusion in the study. The TAO group consisted of patients with the I73.1 diagnosis code, while the DPVD group consisted of patients with E10–E14 (diabetes mellitus) codes along with I70.2 (lower extremity atherosclerosis) and/or I73.9 (peripheral vascular disease) codes. The medical records of these patients and digital subtraction angiography (DSA), computed tomography angiography (CTA), and/or magnetic resonance angiography (MRA) images were retrospectively examined in detail. To improve data quality and minimize selection and information bias, sequential patient selection was performed using predefined inclusion/exclusion criteria, external laboratory results were not included in the analysis, and outcome assessments were conducted independently of the inflammatory/atherogenic indices. The study design, inclusion/exclusion criteria, and patient flowcharts are shown in Figure 1.

Patients were divided into two main cohorts based on their clinical and angiographic characteristics. The TAO group was defined based on a combination of angiographic findings showing a distal nonatherosclerotic lesion pattern consistent with the classic definition using the Shionoya criteria [15]. To distinguish the TAO phenotype as “purely” diabetic atherosclerotic disease, the presence of diabetes mellitus (DM) was considered an exclusion criterion. This cohort included only patients with symptom onset under 50 years of age, aged 18–50 years at index presentation, active tobacco users, or those who had quit smoking within the last 12 months and had a history or finding of superficial thrombophlebitis. The diagnosis was supported by the absence of significant atherosclerotic plaque/calcification in the aortoiliac and femoropopliteal segments on DSA, CTA, and/or MRA, and the demonstration of distal segmental occlusions and a typical “corkscrew” collateral appearance. Of the 127 patients diagnosed with TAO during the study period, seven were excluded: those with diabetes, those without tobacco exposure or whose exposure could not be reliably documented, those with a pattern of widespread multilevel atherosclerotic lesions, and those with findings suggestive of systemic autoimmune/vasculitic disease, hypercoagulable state, or cardioembolic source. The final TAO cohort consisted of 120 patients with a non-diabetic, non-atherosclerotic distal vasculitis pattern of the arteries. As tobacco use is a mandatory component of the TAO diagnostic criteria, smoking status was considered an integral part of the group definition rather than an independent covariate in the statistical analysis. Similarly, because the presence of DM was defined as a mandatory inclusion criterion for DPVD and an absolute exclusion criterion for TAO in the study design, the two cohorts were not compared with respect to DM.

In the DPVD cohort, 423 consecutive patients with a diagnosis of type 1 or type 2 DM for at least 1 year according to the American Diabetes Association (ADA) criteria [16] and ≥50% stenosis or occlusion in at least one infrapopliteal and/or pedal artery in the index extremity detected by BTA, MRA, and/or DSA were screened for inclusion. Patients who did not show clinical/angiographic features favoring TAO, had a lesion pattern consistent with atherosclerotic distribution, and whose records were accessible formed the candidate DPVD pool. After excluding 17 patients due to missing blood counts or lipid profiles, concurrent significant systemic infection, inadequate distal vessel imaging, lack of follow-up for primary endpoints (major amputation, mortality), or reclassification in favor of non-atherosclerotic vasculitis upon reevaluation, the final DPVD cohort comprised 395 patients. DPVD cases were phenotyped according to lesion level. Patients with ≥50% lesions only at the infrapopliteal/pedal level and no significant stenosis in the proximal segments were defined as isolated distal DPVD (d-DPVD), while those with significant stenosis at both the proximal and distal levels in the same extremity were defined as “multilevel disease.” The typical angiographic pattern in favor of TAO was defined as the coexistence of distal segmental occlusions without significant atherosclerotic plaque/calcification in the proximal (aorto-iliac, femoropopliteal) segments and the characteristic “corkscrew” collateral vessel appearance [17]. The d-DPVD subcohort, which showed the highest phenotypic similarity to TAO, was compared with the TAO group in advanced analyses in terms of systemic inflammatory and atherogenic indices, demographic characteristics, ulcer phenotype, extremity involvement pattern, amputation rates, and hospitalization rates.

The common exclusion criteria for both groups were age < 18 years, history of acute arterial embolism or traumatic arterial injury, active malignancy within the last 5 years, active infection/sepsis, major surgery within the last 3 months, autoimmune vasculitis/Takayasu arteritis/collagen tissue disease, acute myocardial infarction or stroke within the last month, advanced renal failure (eGFR < 15 mL/min/1.73 m^2^ or dialysis) [18], and advanced liver failure [18]. Patients with hematologic malignancies/myeloproliferative disease, those receiving long-term systemic corticosteroids (>10 mg/day prednisone equivalent, >3 months) [8], and those receiving other immunosuppressive therapies were also excluded, as these could potentially alter hematologic-derived inflammatory indices. In both cohorts, all consecutive patients who met the diagnostic criteria were included in the study, and no additional subsampling was performed to minimize the selection bias arising from patient selection.

### 2.3. Data Collection, Monitoring, and Endpoints

Demographic, clinical, and laboratory data for patients meeting the inclusion criteria were retrospectively collected from the hospital information management system (HIMS) and national e-health records (e-Nabız). All information was systematically recorded in a predefined standard case report form, which comprehensively reviewed discharge summaries, outpatient notes, surgical reports, radiology/angiography archives, and laboratory records.

**Figure 2:**
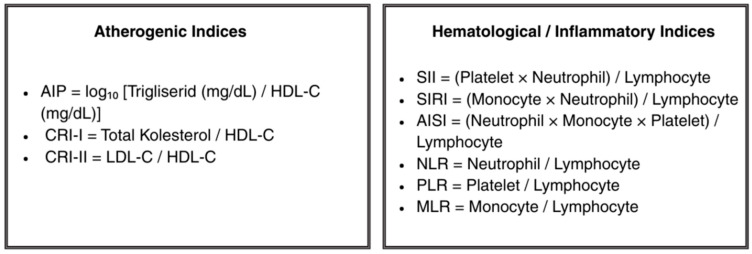
Calculation formulas for hematology-derived systemic inflammatory indices and lipid-derived atherogenic indices applied in this study.

To minimize selection bias, sequential patient selection was performed using predefined inclusion and exclusion criteria. External laboratory results were not used, and outcomes were assessed independently of the inflammatory/atherogenic indices. The index date for each patient was defined as the date of the first diagnostic angiography for lower extremity peripheral artery disease or the date of the first concomitant revascularization; all clinical, imaging, treatment, and laboratory variables were time-locked to this date for the analysis. For the baseline laboratory assessment, measurements taken closest to the index date or during the most clinically stable period before amputation were used. When multiple suitable measurements were available, the value closest to the index date was selected. Inflammatory indices were calculated from absolute cell counts in the complete blood counts, whereas atherogenic indices were calculated from serum lipid profiles, including total cholesterol, LDL-C, HDL-C, and triglyceride levels, using standard formulas (Figure 2).

The follow-up period was defined as the time elapsed from the index date to the study end date or date of death. The dates of major/minor amputations and other vascular events were obtained from the HBYS and radiology/angiography archives, and mortality information was verified using the HBYS and e-Nabız. Patients whose vital status or amputation information could not be obtained at the end of follow-up were considered alive (censored) based on their status on the last recorded date.

Comorbidities included hypertension, hyperlipidemia, coronary artery disease, cerebrovascular disease, chronic kidney disease, COPD, and other chronic systemic diseases. The presence of comorbidity was considered “current” if it was documented in medical records as a physician diagnosis and/or if long-term medication use specific to the disease was found in the records related to the index episode. Previous vascular interventions and medical treatments administered during the index episode (antiplatelet, anticoagulant, statin, cilostazol, iloprost, and other relevant agents) were recorded as distinct variables.

Exposure to cigarettes and other tobacco products was classified into five categories based on patient statements and file records:

- Active smoker: Patients who were still using cigarettes/tobacco products during the index period
- Former recent smoker: Those who quit smoking ≤12 months prior to the index date
- Former remote smoker: Those who quit smoking >12 months prior to the index date
- Never: Patients with no history of regular cigarette/tobacco use
- Unknown: Patients with insufficient information regarding their smoking history

Cigarette exposure was quantitatively assessed using three variables: daily cigarette consumption (pack/day; 1 pack = 20 cigarettes), total smoking duration (years), and pack-year value, which indicates cumulative cigarette exposure [pack-year = pack/day × years]. Pack-years was treated as a continuous variable in statistical analyses. Since tobacco use was a mandatory component of the diagnostic criteria in the TAO cohort, all cases in this group consisted of active or recent quitters; therefore, smoking was evaluated as a structural component of the group variable rather than as an independent covariate in the primary analyses.

Findings related to clinical presentation were obtained from patient files and digital records, consistent with the retrospective design of the study; therefore, symptom duration, Rutherford (0–6) classification, presence of ischemic tissue loss (ulcer/gangrene), affected extremity (right/left/bilateral; lower/upper), and history of superficial thrombophlebitis could only be assessed in patients with sufficient documentation. In patients without photographic records of ulcerative lesions, ulcer characteristics and grading were retrospectively reconstructed from outpatient notes and admission records of the patients. In patients with diabetic foot ulcers/tissue loss, ulcer/necrosis localization was classified into four categories based on the recorded anatomical region: (1) toe/forefoot/dorsum of the foot, (2) plantar pressure areas (metatarsal heads, sole of the foot, and heel), (3) ankle and above, and (4) multiple ulcerations (simultaneous lesions in more than one anatomical region). In cases with multiple ulcers, the most clinically severe lesion was included in the analysis. Patients with no or insufficient information regarding ulcer localization were excluded from the relevant subanalyses.

The modality used for imaging and angiographic evaluation (DSA, BTA, MRA), the anatomical level of the lesions (aorto-iliac, femoropopliteal, infrapopliteal/tibioperoneal, pedal/digital), the degree of stenosis/occlusion, and, when reportable, the collateral vessel status were systematically recorded. DPVD and TAO phenotyping were performed using these variables (definitions in Section 2.2).

The primary endpoints of the study were major lower extremity amputation and all-cause mortality. Major amputation was classified as amputation at or above the ankle level (transtibial, transfemoral, etc.), and amputations at the foot and toe levels were classified as minor amputations. The secondary endpoints were defined to include minor amputation, presence and location of ischemic ulcer/tissue loss, extremity involvement pattern (unilateral/bilateral), and frequency of hospitalization due to lower extremity ischemia.

### 2.4. Statistical Analysis

Continuous variables following a normal distribution are presented as mean ± standard deviation, and categorical variables are presented as counts and percentages. The distribution of continuous variables was assessed using the Kolmogorov–Smirnov/Shapiro–Wilk test and histograms. Student’s *t*-test was used for comparisons between groups for normally distributed variables, the Mann–Whitney U test for non-normally distributed variables, and the chi-square or Fisher’s exact test for categorical variables when necessary.

To examine the relationship between the TAO/DPVD groups and inflammatory/atherogenic markers, univariate logistic regression was first performed; variables with *p* < 0.05 were included in the multivariate model. Multivariate models were constructed using the Forward Likelihood Ratio method, odds ratios (OR) were reported with 95% confidence intervals, and model fit was assessed using the Hosmer–Lemeshow test. ROC curve analysis was performed to determine the discriminatory power of indices showing significant differences between TAO and DPVD; results were given as AUC, and optimal cutoff values were determined based on the Youden index. The sensitivity, specificity, and positive and negative predictive values were calculated at these thresholds. Due to the study design, tobacco exposure was a mandatory component of the diagnostic criteria in the TAO group; therefore, cigarette smoking was not included in the model as an independent variable. All tests were considered statistically significant at *p* < 0.05.

Statistical analyses were performed using the Statistical Package for the Social Sciences (SPSS) version 27.0 (IBM Corp. Armonk, NY, USA). Tables were created using Microsoft^®^ Excel^®^ for Microsoft 365, graphs and images using GraphPad Prism v10.6.1 and Microsoft Visio Professional 2019, and flowcharts were created using Canva Pro.

## 3. Results

The demographic and clinical characteristics of both groups are shown in Table 1. Patients with DPVD/d-DPVD were significantly older than those in the TAO cohort (61.1 ± 12.1 vs. 39.7 ± 7.9 years, median 63 vs. 42; *p* < 0.001), whereas the sex distribution showed a male predominance in both groups (male ratio 84.8% in DPVD, 94.2% in TAO; *p* = 0.08). Body weight and body mass index were similar between the two groups. Classic cardiovascular risk factors were significantly higher in the DPVD cohort than in the TAO cohort. The prevalence of hypertension was 25.1% in DPVD and 15.8% in TAO (*p* = 0.035), and hyperlipidemia was detected at rates of 29.4% and 12.5%, respectively (*p* < 0.001). Chronic kidney disease was more prevalent in the DPVD group (19.7% vs. 10.0%; *p* = 0.013), whereas the prevalence of COPD was similar between the groups (7.3% vs. 5.8%; *p* = 0.57). 68.3% of patients were active smokers, 31.7% had quit smoking within the previous 12 months, and there were no patients who had never smoked. Quantitative smoking burden measures also indicated higher tobacco exposure in favor of TAO: daily cigarette consumption was 1.06 ± 0.45 packs/day in the TAO group and 0.33 ± 0.58 packs/day in the DPVD group (*p* < 0.001); smoking duration was 13.7 ± 8.4 vs. 6.8 ± 11.9 years (*p* < 0.002), and cumulative cigarette exposure (pack-years) was 14.3 ± 11.0 vs. 7.1 ± 14.4 (*p* < 0.003).

DM and “ever-smoker” status were the inclusion/exclusion criteria for the diabetic distal PAD and TAO cohorts, respectively, as part of the study design; no statistical comparisons were made for these lines: NA: not applicable (by study design). Medical and interventional approaches differed significantly between the TAO and DPVD groups (Table 2). Patients with TAO were more frequently followed up with conservative/medical treatment alone (79.2% vs. 61.3%; *p* < 0.001), while revascularization was approximately twice as common in DPVD (35.4% vs. 14.2%), and surgical intervention was particularly more common in this group (11.1% vs. 2.5%; *p* < 0.001). Antiplatelet therapy (98.2% vs. 87.5%; *p* < 0.001) and statin use (45.3% vs. 24.2%; *p* < 0.001) were higher in the DPVD group, while cilostazol use was similar (*p* = 0.091); in contrast, intravenous iloprost therapy was more frequently preferred in the TAO cohort (25.8% vs. 14.4%; *p* = 0.004) (Table 2).

**Figure 3:**
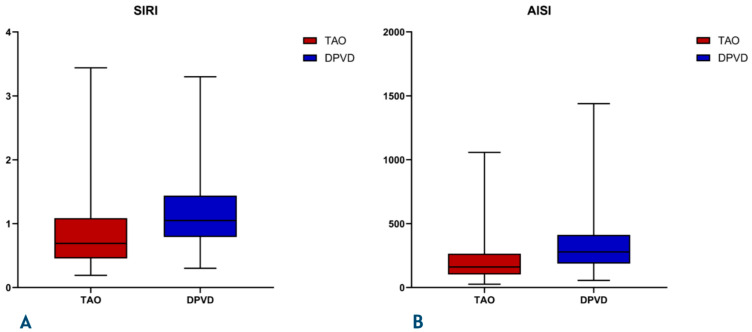
(**A**) Box-plots comparing systemic inflammatory response index (SIRI) and (**B**) aggregate inflammatory systemic index (AISI) between patients with thromboangiitis obliterans (TAO) and diabetic peripheral vascular disease (DPVD). Boxes represent the interquartile range (IQR), horizontal linesindicate the median, and whiskers denote the minimum and maximum values.

**Figure 4:**
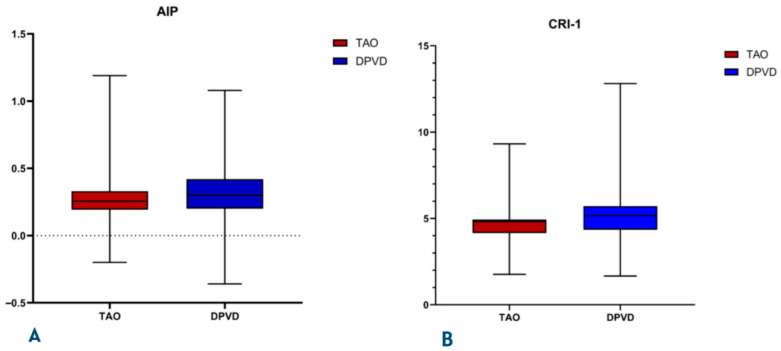
Box-plots comparing lipid-derived atherogenic indices between patients with thromboangiitis obliterans (TAO) and diabetic peripheral vascular disease (DPVD). The left panel (**A**) depicts the Atherogenic Index of Plasma (AIP), while the right panel (**B**) shows Castelli Risk Index-I (CRI-I). Boxes represent the interquartile range (IQR), the central line indicates the median, and whiskers denote the minimum and maximum values, excluding outliers.

The DPVD cohort had a significantly more inflammatory and atherogenic biochemical profile than the TAO cohort (Table 3, Figure 3 and Figure 4). Leukocyte, neutrophil, and monocyte counts were higher in the DPVD group (e.g., WBC 9.3 ± 3.1 vs. 7.3 ± 2.5 × 10^9^/L; *p* < 0.001), and the composite inflammatory indices AISI, SII, SIRI, and NLR were all increased in favor of this group (all *p* < 0.001); PLR was similar. Hemoglobin and hematocrit values were higher in TAO (14.9 ± 2.4 vs. 11.9 ± 2.5 g/dL and 42.0 ± 7.6 vs. 37.8 ± 7.7%; both *p* < 0.001). The lipid profile in patients with DPVD was characterized by higher total cholesterol and LDL-C levels (161.0 ± 39.5 vs. 148.0 ± 37.6 and 111.6 ± 24.9 vs. 96.4 ± 23.9 mg/dL; *p* ≤ 0.004) and lower HDL-C levels (32.2 ± 8.2 vs. 35.4 ± 8.0 mg/dL; *p* < 0.001). In parallel, the AIP, CRI-I, and CRI-II values were also significantly higher (all *p* ≤ 0.001)The composite inflammatory indices SIRI and AISI were systematically higher in the DPVD group than in the TAO group (all *p* < 0.001; Table 3) The distributions of systemic inflammatory and atherogenic indices between the two groups are illustrated in Figure 3 and Figure 4. Specifically, SIRI and AISI are presented in box-and-whisker plots in Figure 3A and Figure 3B, respectively, whereas AIP and CRI-1 are displayed in Figure 4A and Figure 4B. Urea and creatinine levels were higher in DPVD (*p* < 0.001), while CRP showed only a borderline increase (*p* = 0.066).

**Figure 5:**
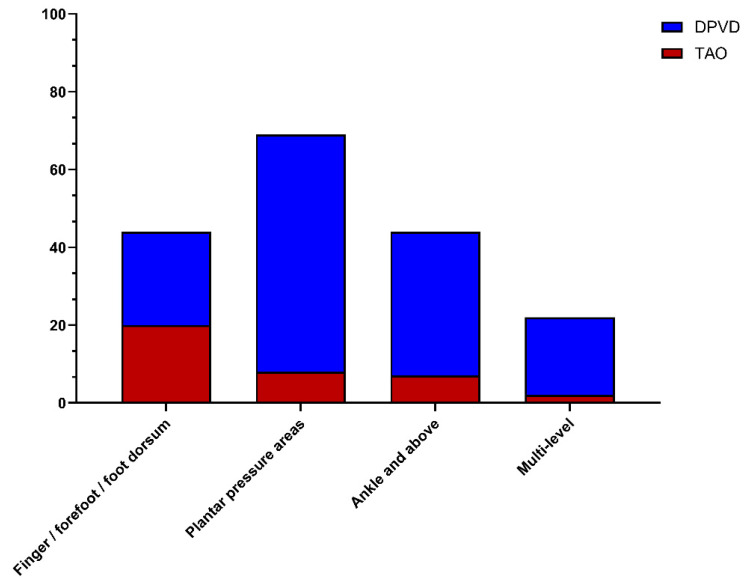
Anatomical distribution of ischemic ulcer locations in TAO and diabetic distal PAD patients.

**Figure 6:**
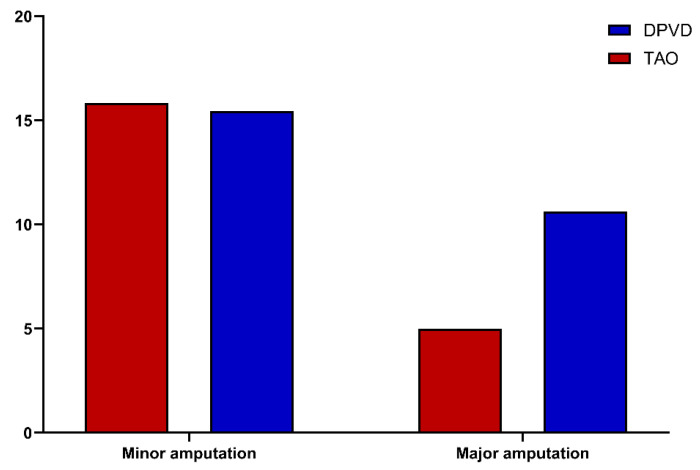
Rates of minor and major lower limb amputation in TAO and diabetic distal PAD.

According to the clinical outcomes summarized in Table 4, the prevalence of active ischemic ulcers was similar in the TAO and DPVD groups (30.8% and 35.9%, respectively; *p* = 0.303). However, ulcer topography differed significantly: in DPVD, lesions were predominantly located in plantar pressure areas and the ankle/above the ankle, whereas in TAO, distal acral ulcers (toe/forefoot/dorsum of the foot) were more common (16.7% vs. 6.1%; *p* < 0.001; Figure 5). The pattern of limb involvement also differed; the disease was mostly limited to a single limb in patients with DPVD (87.8%), whereas most cases of TAO involved bilateral or multiple limbs (71.7%; *p* < 0.001). Although the total amputation rates were similar (TAO, 21.7%; DPVD, 26.6%; *p* = 0.279), major amputation was more common in the DPVD group (11.1% vs. 5.0%; *p* = 0.047), whereas minor amputation and re-amputation rates remained comparable (15.4% vs. 16.7%, respectively; *p* = 0.918 and 8.0% vs. 7.5%; *p* = 0.871; (Figure 6). Although the rate of at least one hospital admission was similar, the number of admissions per patient was higher in the DPVD group (1.01 ± 1.65 vs. 0.54 ± 1.00; *p* = 0.047). During approximately 8–9 months of follow-up, all-cause mortality was significantly higher in the DPVD group than in the TAO group (8.1% vs. 2.5%; *p* = 0.033).

When comparing the d-DPVD (infrapopliteal/pedal) subgroup with the TAO cohort, patients with d-DPVD were significantly older (63.6 ± 11.1 vs. 39.9 ± 8.2 years; *p* < 0.01). The systemic inflammatory burden was markedly increased, the and SII, SIRI, AISI, and NLR values were significantly higher in the d-DPVD group than in the TAO group (all *p* ≤ 0.001). Similarly, the atherogenic indices, such as AIP, CRI-I, and CRI-II, were also higher in d-DPVD (all *p* ≤ 0.01). Clinically, the prevalence of active ischemic ulcers was higher in the d-DPVD group than in the TAO group (48.8% vs. 30.8%; *p* = 0.002). The pattern of limb involvement differed; in d-DPVD cases, the disease was mostly limited to a single limb, whereas a significant proportion of patients with TAO had bilateral or multiple limb involvement (*p* < 0.01). Furthermore, the rate of at least one hospitalization was significantly higher in the d-DPVD group than in the TAO group (53.1% vs. 30.8%; *p* < 0.01; Table 5).

**Figure 7:**
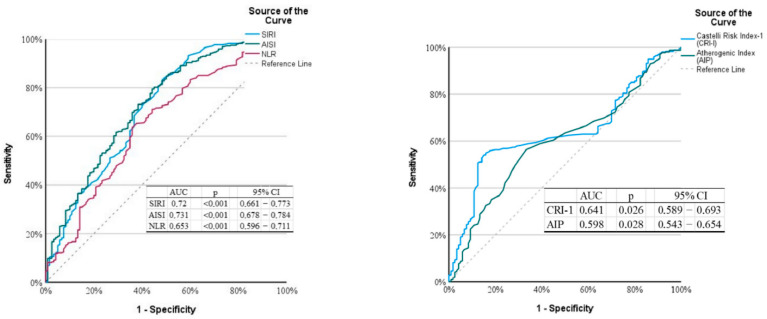
Receiver operating characteristic curves of systemic inflammatory and atherogenic indices for discriminating diabetic distal PAD from thromboangiitis obliterans.

In the logistic regression analysis, group status (DPVD = 1, TAO = 0) was considered as the dependent variable (Table 6). In univariate analyses, advanced age, presence of hypertension and hyperlipidemia, lower pack-years, high inflammatory indices (NLR, SII, SIRI, AISI), and atherogenic indices (AIP, CRI-I) were significantly associated with DPVD (all *p* < 0.05). In the multivariate Forward LR model, only age, pack-years, SIRI, and CRI-I remained as independent predictors: each additional year of age increased the likelihood of having diabetic DPVD by approximately 16% (OR 1.16; 95% CI 1.13–1.20; *p* < 0.001), a higher cumulative smoking burden was inversely associated with TAO (OR 0.97; 95% CI 0.95–0.99; *p* = 0.001), and SIRI, as a strong indicator of systemic inflammation, increased the likelihood of DPVD approximately 4.5-fold (OR 4.51; 95% CI 2.22–9.14; *p* < 0.001), and CRI-I was identified as an independent predictor of atherogenic burden (OR 1.32; 95% CI 1.05–1.64; *p* = 0.016). In the ROC analysis, inflammatory indices provided moderate discrimination in the TAO–DPVD distinction (Figure 7): The AUCs for AISI and SIRI were 0.731 (95% CI 0.678–0.784; *p* < 0.001) and 0.720 (95% CI 0.661–0.773; *p* < 0.001), respectively, and were superior to NLR (AUC 0.653). The AUC values for the atherogenic indices were more limited (Figure 6): 0.641 (95% CI 0.589–0.693; *p* = 0.026) for CRI-I and 0.598 (95% CI 0.543–0.654; *p* = 0.028) AIP.

Optimal cutoff points were determined for the parameters with the highest discriminatory power to distinguish TAO from DPVD using ROC analysis (Table 7). Age was the strongest predictor alone, with an AUC of 0.923 (95% CI 0.901–0.946; *p* < 0.001), a sensitivity of 81.8%, and a specificity of 97.5% at a threshold of >50.5 years. Systemic inflammatory indices showed moderate discrimination: for SIRI, at a cutoff of >0.695, the AUC was 0.717 (95% CI 0.661–0.773; *p* < 0.001; sensitivity 84.1%, specificity 50.8%), and for AISI, the AUC was 0.731 (95% CI 0.678–0.784; *p* < 0.001; sensitivity 79.5%, specificity 55.8%) at a value >177.1. The discriminatory power of the atherogenic indices was more limited; for CRI-I, the AUC was 0.641 (95% CI 0.589–0.693; *p* = 0.001; sensitivity 54.1%, specificity 85.0%), and for AIP, an AUC of 0.598 (95% CI 0.543–0.654; *p* < 0.001; sensitivity 58.4%, specificity 63.3%) was obtained at a threshold of >0.285.

## 4. Discussion

**Figure 8:**
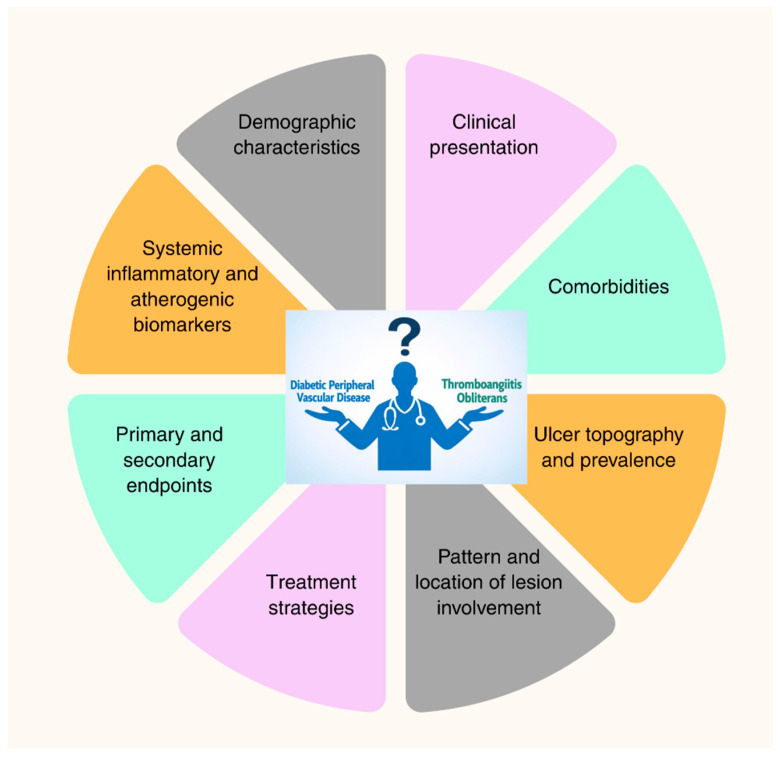
Multidimensional framework illustrating key clinical, biological, and outcome domains differentiating diabetic peripheral vascular disease and thromboangiitis obliterans.

This study compared the non-diabetic TAO cohort representing Buerger’s disease with the DPVD cohort, not only in terms of systemic inflammatory and atherogenic indices; demographic characteristics, classic cardiovascular risk factors, ulcer phenotype, extremity involvement pattern, medical/surgical treatments administered, amputation, mortality, and frequency of hospital admissions. Furthermore, the “isolated distal” d-DPVD subgroup, which phenotypically resembles TAO most closely, was defined and compared with TAO in a separate subgroup analysis. To support a comprehensive interpretation of the findings. Figure 8 summarizes the distinguishing features of DPVD and TAO in terms of clinical, biological, and outcome dimensions.

One of the most fundamental findings of our study was the presence of distinct demographic and risk factor differentiation between TAO and DPVD. As expected, the TAO cohort was characterized by younger age, marked male predominance, and high cumulative tobacco exposure, presenting a relatively “cleaner” profile in terms of atherosclerotic risk factors such as DM, hypertension, hyperlipidemia, and chronic kidney disease. This profile reflects the “classic Buerger phenotype” of TAO, defined as tobacco-related nonatherosclerotic distal segmental inflammatory arteriolitis [19]. In contrast, DPVD represents an atherosclerotic phenotype characterized by advanced age and intense cardiometabolic comorbidity, developing against a background of chronic hyperglycemia, oxidative stress, endothelial dysfunction, and progressive macro-microangiopathy, compounded by systemic inflammation, dyslipidemia, and renal dysfunction [20]. Multiple large cohorts in the literature have demonstrated a significant increase in the risk of major amputation and mortality in PVD associated with diabetes [21,22] and the higher rates of major amputation and all-cause mortality observed in the DPVD group compared to the TAO group in our study support this high-risk profile. These findings indicate that DPVD should be considered not only as an extremity-threatening but also as a life-threatening vasculopathy, and that limb- and life-saving strategies should be applied more aggressively and systematically in this group.

Our study demonstrated that TAO and DPVD are significantly different in terms of biochemistry, and that both the systemic inflammatory burden and atherogenic profile are significantly elevated in DPVD. Although TAO is classically defined as “inflammatory vasculitis,” the higher detection rates of SIRI, AISI, and related indices in DPVD appear paradoxical at first glance. This situation should be interpreted as a hematological/biochemical reflection of the diabetes-specific axis of chronic low-grade inflammation, immune dysregulation, and atherogenic dyslipidemia [23]. These findings are consistent with the growing evidence in the literature that systemic immune-inflammatory indices (SII, SIRI, and NLR) are associated with peripheral artery disease incidence, diabetic foot ulcers (DFU), and adverse cardiovascular outcomes in diabetes [13,24,25]. These composite cellular indices, which combine a neutrophil/monocyte-dominant proinflammatory response and a relative decrease in the lymphocyte fraction into a single scale, may represent chronic vascular inflammation more sensitively than individual markers under stable disease conditions [26]. In this context, our findings expand the current evidence framework by demonstrating that SIRI and AISI show moderate discriminatory performance not only in prognostic predictions but also in distinguishing two pathobiologically distinct peripheral vasculopathies (TAO and DPVD). The significantly higher levels of NLR, SIRI, and AISI in patients with DPVD compared to those with TAO highlight the extent of chronic “low-grade” inflammation caused by diabetes [27]. Hyperglycemia, oxidative stress, advanced glycation end products (AGEs), endothelial dysfunction, and secondary infection burden accompanying foot ulcers may be the possible mechanisms underlying this increased inflammatory response [28,29]. In contrast, inflammation in TAO is a segmental, vascular bed-localized immunothrombotic process triggered by tobacco, unlike in DPVD [19]. A similar differentiation was observed for the lipid parameters. Significantly higher AIP and Castelli risk indices (CRI-I/II) in DPVD and the independent prediction of the diabetic phenotype by CRI-I in multivariate analysis indicate that DPVD develops in a distinctly atherogenic lipid environment, whereas in TAO, a more benign lipid profile and low CRI values confirm the non-atherosclerotic nature of the disease at the biochemical level [30,31]. Furthermore, the persistence of high AIP/CRI levels even in the d-DPVD subgroup suggests that this phenotype may be a distal reflection of systemic atherogenic/inflammatory burden rather than a “TAO-like benign distal variant.” Clinically, this indicates that in DPVD, aggressive lipid-lowering therapy and comprehensive cardiovascular risk reduction should be the primary approach, even in the presence of infrapopliteal involvement alone, whereas lipid-focused strategies should remain secondary in TAO. Finally, although CRP showed a non-significant increase in DPVD, the detection of a marked difference in composite indices integrating cellular components supports the view that chronic low-grade vascular inflammation in stable PAD can be more sensitively captured by cell-based indices rather than by individual acute-phase reactants [13].

The ulcer topography and extremity involvement patterns identified in our study revealed the distinct clinical manifestations of phenotypic differentiation between TAO and DPVD. In DPVD, ulcers are localized primarily at plantar pressure points and in the ankle/proximal segments under the interaction of peripheral neuropathy, biomechanical pressure, and ischemia, consistent with the literature on diabetic foot [32]. In TAO, ulcers are predominantly observed in the distal acral regions (toes, forefoot, and dorsum of the foot) and are frequently associated with bilateral or multiple limb involvement [19]. This distribution can be considered a distinctive clinical signature reflecting tobacco-induced, segmental, non-atherosclerotic distal vasculitis and a “corkscrew” collateral pattern in TAO [33] and neuroischemic tissue loss due to the interaction of neuropathic pressure areas with macro- and microangiopathy in DPVD [34]. Similarly, the fact that the disease is mostly limited to a single extremity in DPVD, whereas it involves bilateral or multiple extremities in TAO, supports the systemic segmental vasculitis nature of TAO and the regionally severe but distinct atherosclerotic and diabetic damage-based phenotypes of DPVD.

Another key finding of our study is that the clinical outcomes were significantly worse in favor of DPVD. Although the overall amputation rate appeared similar between the two groups, major amputation and all-cause mortality were significantly higher in the DPVD group, along with more frequent hospital admissions, indicating that this phenotype carries a high-risk profile in terms of both limb- and life-threatening outcomes. This finding is consistent with recent cohort data demonstrating an increased risk of amputation and mortality in patients with diabetes and chronic limb-threatening ischemia compared with non-diabetic populations [35]. In contrast, TAO, despite carrying a substantial risk of distal ulceration and amputation, appears to represent a relatively more benign form of limb ischemia, characterized by lower short- to mid-term mortality and the potential for partial remission with smoking cessation alone. In DPVD, the risk of limb loss due to peripheral ischemia, combined with cardiovascular mortality driven by a widespread atherosclerotic burden, suggests that disease involvement seemingly confined to the infrapopliteal segment actually reflects advanced systemic metabolicvascular damage.

In patients with critical limb ischemia managed nonoperatively, autoamputation represents an infrequent but clinically relevant outcome, particularly in cases with distal, limited, and non-infected ischemic lesions [36]. Autoamputation should not be considered an active therapeutic strategy but rather a potential disease course observed in carefully selected patients in whom revascularization is technically unfeasible or clinically inappropriate under close surveillance [37]. The clinical implications of autoamputation differ substantially between patients with TAO and those with DPVD. In TAO, the predominantly distal and segmental ischemic pattern, relative preservation of proximal arteries, and development of collateral circulation may allow spontaneous demarcation and separation of limited acral necrosis in selected cases [38]. Conversely, in DPVD, autoamputation is more frequently associated with uncontrolled tissue loss, secondary infection, and sepsis due to diffuse microvascular dysfunction and impaired wound healing, and is therefore generally regarded as an unfavorable outcome rather than an acceptable alternative to intervention [39]. Accordingly, the prognostic and clinical significance of autoamputation appears to be strongly dependent on the underlying disease phenotype, ischemic distribution, and comorbidity. Our findings support the interpretation that autoamputation should be evaluated within a disease-specific and patient-centered context rather than as a uniform indicator of treatment success or failure in nonoperatively managed ischemic limbs.

Our study indicates that treatment strategies are also distinctly different between the two phenotypes in a manner consistent with their pathobiology and anatomical involvement. The DPVD cohort exhibited more intensive antiplatelet and statin use and higher revascularization rates. This situation can be considered a reflection of a more guideline-based approach [40]. The more frequent selection of revascularization in DPVD can be explained by the clinical benefit of interventional treatment applied to anatomically suitable segments in the presence of widespread atherosclerotic macrovascular involvement. In contrast, the management of TAO is predominantly conservative/pharmacological, involving vasodilators and prostacyclin analogs (e.g., iloprost) [41] which can be attributed to the limited technical feasibility and long-term patency of revascularization in the small vessel-predominant, non-atherosclerotic, and immune-mediated distal vasculitis patterns of the disease [17,37,42]. Therefore, in TAO, an approach focused on absolute smoking cessation, aggressive risk factor control, and pharmacotherapy, as emphasized in the current guidelines, appears rational [40]. In contrast, treatment priorities for DPVD should focus on pressure reduction, infection control, optimization of metabolic and lipid regulation, and timely revascularization in appropriate cases [43]. Furthermore, the fact that DPVD exhibits a more aggressive clinical spectrum than TAO, threatening both limb loss and life, even when involvement is limited to the infrapopliteal/pedal segment, supports the notion that the two diseases differ not only in anatomical distribution but also in systemic risk burden and treatment integration requirements. Thus, TAO represents a vasculitis-focused treatment paradigm supported by targeted revascularization in selected cases, whereas DPVD represents a widespread atheromatous disease spectrum requiring systemic risk reduction integrated with revascularization at all levels.

One of the most original aspects of this study is the direct comparison between TAO and the anatomically closest isolated distal DPVD (d-DPVD) subgroups. Despite similar infrapopliteal and pedal distributions, patients with d-DPVD were significantly older and exhibited markedly higher systemic inflammatory and atherogenic burdens. Furthermore, its characterization by a more severe clinical course (more frequent ulcers, amputations, and hospitalizations) suggests that the DPVD phenotype is determined more by the global cardiometabolic risk burden than by the anatomical level [39]. Despite the relatively low atherogenic burden in TAO, the frequent occurrence of multiple limb involvement reinforces its systemic vasculitic character [8], whereas in d-DPVD, it reinforces the need for clinically aggressive risk factor modification and close monitoring.

In the multivariate logistic regression analysis, age, cumulative smoking load (pack-years). SIRI, and CRI-I were identified as independent predictors for distinguishing TAO from DPVD. Although high discriminatory power was observed for age in the ROC analyses, it should be noted that this is partly a reflection of age and diabetes restrictions in the study design. In contrast, the moderate discriminative performance of SIRI/AISI and the more limited performance of CRI-I/AIP suggest that these indices can be used as supplementary but auxiliary biomarkers in addition to classic clinical variables. In particular, in borderline cases where clinical and angiographic patterns overlap, high SIRI and CRI-I values, consistent with widespread systemic inflammation in DPVD and more localized immunothrombotic processes in TAO, may provide complementary information supporting the underlying pathophysiology.

## 5. Study Limitations

This study had several important limitations. First, despite its retrospective and single-center design, sequential case selection, and standardized data extraction, it does not completely eliminate the risk of selection and information bias due to confounding factors. The exclusion of non-respondents, cases with incomplete records, and cases followed at external centers is a potential source of selection bias.

Some variables related to clinical presentation (duration of symptoms, Rutherford/Fontaine stage, detailed ulcer classification, peripheral neuropathy measurements, etc.) could not be adequately documented in all patients and were therefore only partially reflected in the multivariate models. Ulcer location and clinical stage were retrospectively coded from discharge summaries and examination notes without photographic documentation in most cases, increasing the likelihood of information bias in the study.

Inflammatory and atherogenic indices were derived from laboratory measurements at a single point in time, considered clinically “stable,” and time-dependent variability, subclinical infections, concomitant inflammatory conditions, medication use (statins, anti-inflammatory agents), and glycemic control levels could not be fully controlled.

The relatively short follow-up period represents an important limitation of this study and should be considered when interpreting the prognostic outcomes. Although early events such as major amputation, hospitalization, and short-term mortality were adequately captured, long-term limb-related and cardiovascular outcomes may not be fully reflected, particularly in patients with TAO with a potentially more indolent disease course. Accordingly, the observed differences in prognostic endpoints primarily reflect short- to mid-term risk, and caution is required when extrapolating these findings to long-term outcomes of the study.

The diagnosis of TAO based solely on clinical-angiographic criteria does not completely rule out the possibility of misclassifying atypical atherosclerotic or other vasculitis cases without histopathological confirmation. Furthermore, the upper age limit and exclusion of diabetes in TAO and the mandatory criterion of diabetes in DPVD, by design, pull the age and comorbidity profile to two extremes, partially inflating the discriminatory power of age and classic risk factors and limiting the evaluation of “intermediate” phenotypes.

Although treatment strategies were recorded in detail, treatment adherence, dose–response relationships, and the comparative efficacy of different approaches could not be evaluated due to the retrospective observational design. Finally, the fact that the patient population belonged to a tertiary center with a high burden of smoking-related vasculitis and diabetes may limit the generalizability of the findings to different healthcare systems and populations.

## 6. Conclusions

The findings of this study indicate that TAO and DPVD are two distinct entities, both biologically and clinically, despite overlapping distal ischemic presentations. Our results emphasize that the anatomical lesion level alone may be insufficient to explain the disease course and prognosis; conversely, the systemic inflammatory-atherogenic burden and clinical phenotype play decisive roles. In this context, composite indices such as SIRI and AISI, derived from routine laboratory parameters, can serve as practical tools to support differential diagnosis and risk classification when evaluated alongside clinical and angiographic data. In clinical practice, these data support aggressive cardiometabolic risk reduction, multidisciplinary diabetic foot management, and prioritization of timely revascularization in DPVD, whereas in TAO, they support a smoking cessation-based approach and phenotype-adapted vasculitis-focused treatment. In the future, prospective validation of these indices in larger and more heterogeneous cohorts and their integration into multiparametric diagnostic/prognostic models could contribute to the development of more personalized strategies for managing ischemia threatening the distal limb by strengthening the risk stratification.

## Figures and Tables

**Table 1:** Comparison of baseline demographic and clinical characteristics between the TAO and diabetic distal peripheral vascular disease (DPVD) cohorts.

		DPVD								TAO							*p* *
			Mean ± SD			Median	Min	−	Max	Mean ± SD			Median	Min	−	Max	
Age			61.05	±	12.10	63.00	18.00	−	80.00	39.67	±	7.86	42.00	19.00	−	49.00	<0.001
Sex																	
	Male		335		85					113		94%					0.08
	Female		60		15					7		6%					
Weight			79.11	±	11.46	77.00	51.00	−	116.00	79.06	±	12.03	79.00	54.00	−	114.00	0.951
Body mass index (BMI)			27.92	±	4.91	26.59	16.55	−	43.85	27.46	±	4.61	26.30	20.18	−	40.37	0.386
Hypertension		*n/%*	99		25.06%					19		15.83%					0.035
DM		*n/%*	395		100.00%					0		0.00					NA
Hyperlipidemia		*n/%*	116		29.37%					15		12.50%					<0.001
Chronic kidney disease		*n/%*	78		19.75					12		10.00%					0.013
COPD		*n/%*	29		7.34%					7		5.83%					0.570
Smoker		*n/%*															
	Never	*n/%*	259		65.57%					0		0.00%					NA
	Active smoker	*n/%*	79		20.00					82		68.33					
	Former recent smoker	*n/%*	25		6.33%					38		31.67					
	Former remote smoker	*n/%*	32		8.10					0		0.00%					
Daily cigarette use (pack/day)			0.33	±	0.58	0.00	0.00	−	3.00	1.06	±	0.45	1.00	0.00	−	3.00	<0.001
Duration of smoking (years),			6.77	±	11.94	0.00	0.00	−	50.00	13.66	±	8.37	12.00	0.00	−	42.00	<0.002
Smoking burden (pack-years)			7.12	±	14.39	0.00	0.00	−	123.00	14.25	±	10.98	11.25	0.00	−	60.00	<0.003

DPVD, Diabetic peripheral vascular disease; TAO, thromboangiitis obliterans; SD, standard deviation; DM, diabetes mellitus; COPD, chronic obstructive pulmonary disease. *, Statistical significance was defined as a two-tailed *p*-value < 0.05.

**Table 2:** Comparison of medical treatment and revascularization strategies in patients with thromboangiitis obliterans (TAO) and diabetic peripheral vascular disease (DPVD).

			DPVD		TAO		*p*
			*n*	%	*n*	%	
**Type of treatment**							
		Medical treatment	242	61.27	95	79.17%	<0.001
		Revascularization	140	35.44	17	14.17	
**Type of revascularization**							
		Surgical	44	11.14	3	2.50%	<0.001
		Endovascular	79	20.00%	12	10.00	
		Both/hybrid	17	4.30%	2	1.67%	
**Pharmacological treatment**							
	Use of antiplatelet agents		388	98.23	105	87.50%	<0.001
	Antiplatelet type						
		ASA	296	74.94%	86	71.67%	<0.001
		Clopidogrel	68	17.22%	12	10.00	
		Dual antiplatelet therapy	24	6.08%	7	5.83	
	Statin use		179	45.32%	29	24.17%	<0.001
	Cilostazol use		122	30.89%	47	39.17%	0.091
	Iloprost therapy (IV)		57	14.43	31	25.83%	0.004

TAO, thromboangiitis obliterans; DPVD, diabetic distal peripheral vascular disease; SD, standard deviation; ASA, acetylsalicylic acid; IV, intravenous.

**Table 3:** Baseline hematologic, biochemical, inflammatory, and atherogenic indices in TAO and DPVD.

		DPVD				TAO				*p*
		Mean ± SD			Median	Mean ± SD			Median	
**CBC parameters**										
	WBC	9.32	±	3.10	8.92	7.25	±	2.46	6.71	0.000
	Hemoglobin	11.87	±	2.49	12.28	14.88	±	2.43	15.53	0.000
	Hematocrit	37.80	±	7.65	38.90	42.03	±	7.56	42.89	0.000
	Platelet	261.63	±	82.64	240.00	244.23	±	80.94	217.00	0.002
	Neutrophil	4.91	±	1.42	4.75	4.01	±	1.26	3.81	0.000
	Lymphocyte	2.33	±	0.50	2.31	2.32	±	0.58	2.23	0.041
	Monocyte	0.54	±	0.17	0.52	0.43	±	0.16	0.44	0.000
**Systemic inflammatory markers**										
	AISI	327.68	±	196.76	277.90	202.79	±	142.89	160.32	0.000
	SII	564.52	±	288.81	511.37	435.71	±	220.53	391.59	0.000
	SIRI	1.17	±	0.52	1.05	0.82	±	0.49	0.69	0.000
	NLR	2.18	±	0.77	2.07	1.80	±	0.62	1.73	0.000
	PLR	116.12	±	44.09	106.96	108.90	±	36.78	103.81	0.172
	MLR	0.25	±	0.10	0.24	0.20	±	0.08	0.19	0.000
**Atherogenic indices**										
	AIP	0.32	±	0.20	0.30	0.26	±	0.20	0.26	0.001
	CRI-I	5.23	±	1.55	5.17	4.58	±	1.16	4.82	0.000
	CRI-II	3.65	±	1.25	3.46	3.05	±	1.35	2.74	0.000
**Basic biochemistry parameters**										
	Urea	42.17	±	15.91	40.90	31.58	±	8.88	30.90	0.000
	Creatinine	1.15	±	0.29	1.15	0.96	±	0.18	0.96	0.000
	Sodium	138.38	±	4.10	139.00	138.35	±	4.16	138.00	0.854
	Potassium	4.33	±	0.49	4.42	4.24	±	0.59	4.21	0.256
	LDH	295.79	±	94.94	279.00	270.53	±	92.05	252.50	0.006
	AST	30.74	±	25.80	27.00	29.09	±	17.01	25.00	0.283
	ALT	36.73	±	26.27	34.00	38.00	±	23.98	35.70	0.410
	CRP	6.68	±	17.04	1.67	3.57		3.82	3.15	0.066

TAO, thromboangiitis obliterans; DPVD, diabetic distal peripheral vascular disease; WBC, white blood cell (leukocyte); HDL-C, high-density lipoprotein cholesterol; LDL-C, low-density lipoprotein cholesterol; VLDL, very-low-density lipoprotein; AISI, Aggregate Index of Systemic Inflammation; SII, Systemic Immune-Inflammation Index SIRI, Systemic Inflammation Response Index; NLR, neutrophil-to-lymphocyte ratio; PLR, platelet-to-lymphocyte ratio; MLR, monocyte-to-lymphocyte ratio; AIP, Atherogenic Index of Plasma; CRI-I, Castelli Risk Index-I; CRI-II, Castelli Risk Index-II; LDH, lactate dehydrogenase; AST, aspartate aminotransferase; ALT, alanine aminotransferase; CRP, C-reactive.

**Table 4:** Clinical outcomes, amputations, hospitalizations, and mortality in TAO and diabetic distal peripheral artery disease.

			DPVD				TAO				*p*
			Mean	±	Sd	Median	Mean	±	Sd	Median	
**Active ulcer**			142		35.95		37		30.83%		0.303
**Ulcer locations**											
	Distal acral	*n/%*	24		6.08		20		16.67%		<0.001
	Plantar pressure areas	*n/%*	61		15.44%		8		6.67		
	Ankle and above	*n/%*	37		9.37		7		5.83		
	Multiple ulcerations	*n/%*	20		5.06		2		1.67%		
**Extremity involvement**											
	Single extremity	*n/%*	347		87.85		34		28.33%		<0.001
	Bilateral extremities	*n/%*	48		12.15		86		71.67%		
**Amputation**		*n/%*	105		26.58		26		21.67%		0.279
**Re-amputation**		*n/%*	42		10.63%		9		7.50		0.314
**Amputation type**											
	Minor amputation	*n/%*	61		15.44		20		16.67		0.918
	Major amputation	*n/%*	44		11.14		6		5.00%		0.047
**Hospital admission**		*n/%*	146		36.96%		37		30.83%		0.219
**Number of hospital admissions**			1.01	±	1.65	0.00	0.54	±	1.00	0.00	0.047
**Follow-up period (weeks)**			37.23	±	14.29	41.00	33.62	±	15.72	38.50	0.024
**All-cause mortality**		*n/%*	32		8.10		3		2.50%		0.033

TAO, thromboangiitis obliterans; DPVD, diabetic peripheral vascular disease; SD, standard deviation.

**Table 5:** Demographic characteristics, systemic inflammatory/atherogenic indices, and limb-related outcomes in TAO versus isolated distal diabetic PAD.

			d-DPVD (*n* = 162)				TAO (*n* = 120)				*p* *
			Mean ± SD			Median	Mean ± SD			Median	
**Demographic characteristics**											
	Age		63.59	±	11.06	65.00	39.93	±	8.20	42.00	<0.01
	Sex										
		Male	116		71.60%		113		94.17%		<0.01
		Female	46		28.40		7		5.83%		<0.01
	Body mass index (BMI)		27.90	±	4.90	26.59	27.46	±	4.61	26.30	0.489
	DM		162		100.00%		0		0.00%		NA
	Hyperlipidemia		42		25.93%		15		12.50%		0.006
	Hypertension		40		24.69%		19		15.83%		0.071
**Systemic inflammatory markers**			478.06	±	209.98	444.15	435.71	±	220.53	391.59	0.000
	SII		1.07	±	0.45	0.95	0.82	±	0.49	0.69	0.000
	SIRI		277.08	±	134.65	247.14	202.79	±	142.89	160.32	0.000
	AISI		1.95	±	0.65	1.87	1.80	±	0.62	1.73	0.000
	NLR										
**Atherogenic indices**											
	Atherogenic Index (AIP)		0.32	±	0.22	0.32	0.26	±	0.20	0.26	0.001
	Castelli Risk Index-1 (CRI-I)		5.21	±	1.43	5.09	4.58	±	1.16	4.82	0.000
	Castelli Risk Index-1 (CRI-II)		3.52	±	1.25	3.36	3.05	±	1.35	2.74	0.000
**C-reactive protein (CRP)**			9.38	±	24.42	1.60	3.58	±	3.83	3.15	0.066
**Extremity involvement**											
		Single extremity	143		88.27		34		28.33%		<0.01
		Bilateral extremities	20		12.35%		86		71.67%		
**Active ulcer lesion**			79		48.77		37		30.83%		0.002
**Ulcer locations**											
		Distal acral	15		9.26		20		16.67%		0.001
		Plantar pressure areas	40		24.69%		8		6.67		
		Ankle and above	20		12.35		7		5.83		
		Multiple ulcerations	4		2.47%		2		1.67%		
**Amputation outcomes**											
	Amputation		53		32.72%		26		21.67%		0.041
	Re-amputation		13		8.02%		9		7.50%		0.871
	Amputation type										
		Major amputation	19		11.73%		6		5.00		0.069
		Minor amputation	34		20.99%		20		16.67%		
**Hospitalization and healthcare utilization**											
	Hospital admission		86		53.09		37		30.83%		<0.01

TAO, thromboangiitis obliterans; d-DPVD, isolated distal diabetic peripheral vascular disease; DM, diabetes mellitus, AISI, Aggregate Index of Systemic Inflammation; SII, Systemic Immune-Inflammation Index; SIRI, Systemic Inflammation Response Index; NLR, neutrophil-to-lymphocyte ratio; AIP, Atherogenic Index of Plasma; CRI-I, Castelli Risk Index-I; CRI-II, Castelli Risk Index-II; CRP, C-reactive protein. *, Statistical significance was defined as a two-tailed *p*-value < 0.05.

**Table 6:** Univariate and multivariate logistic regression analysis identifying independent determinants of DPVD versus TAO.

		Univariate Model				Multivariate Model (Forward LR)			
		OR	95% C.I.		*p*	OR	95% CI		*p*
Age		1.162	1.130	1.196	<0.001	1.162	1.127	1.197	<0.001
Sex									
	Female (ref)								
	Male	0.346	1.284	6.508	0.010				
BMI		1.020	0.977	1.066	0.362				
Smoking burden (pack-years)		0.968	0.954	0.982	<0.001	0.968	0.949	0.988	0.001
Hypertension		1.778	1.036	3.052	0.037				
Hyperlipidemia		2.910	1.625	5.212	<0.001				
SII		1.002	1.001	1.003	<0.001				
SIRI		5.622	3.207	9.853	<0.001	4.506	2.221	9.143	<0.001
AISI		1.006	1.004	1.008	<0.001				
CRP		1.036	1.002	1.072	0.039				
AIP		4.533	1.542	13.324	0.006				
CRI-I		1.416	1.201	1.669	<0.001	1.316	1.053	1.644	0.016
Active ulcer wound		1.259	0.812	1.952	0.303				
Amputation		1.309	0.803	2.133	0.280				
Re-amputation		1.467	0.693	3.106	0.317				
Amputation Type					0.423				
	Minor amputation	0.971	0.554	1.701	0.918				
	Major amputation	0.677	0.261	1.759	0.423				
All-cause mortality		3.438	1.034	11.433	0.044				

TAO. Thromboangiitis obliterans; DPVD. diabetic peripheral vascular disease; AISI. Aggregate Index of Systemic Inflammation; SII. Systemic Immune-Inflammation Index; SIRI. Systemic Inflammation Response Index; AIP. Atherogenic Index of Plasma; CRI-I. Castelli Risk Index-I; CRP. C-reactive protein.

**Table 7:** Optimal cut-off values and diagnostic performance of clinical, inflammatory, and atherogenic indices for discriminating diabetic distal PAD from TAO.

Variables	AUC	*p*	95% CI		Cut-Off	Sensitivity	Specificity	Positive Predictive Value	Negative Predictive Value
Age	0.92	<0.001	0.90	0.95	50.50	0.82	0.98	0.99	0.62
SIRI	0.72	<0.001	0.66	0.77	0.70	0.84	0.51	0.85	0.49
AISI	0.73	<0.001	0.68	0.78	177.14	0.79	0.56	0.86	0.45
AIP	0.60	0.001	0.54	0.65	0.29	0.58	0.63	0.85	0.30
CRI-1	0.64	0.001	0.59	0.69	5.08	0.54	0.85	0.92	0.36

TAO. Thromboangiitis obliterans; DPVD. Diabetic peripheral vascular disease; SIRI. Systemic Inflammation Response Index; AISI. Aggregate Index of Systemic Inflammation; AIP. Atherogenic Index of Plasma; CRI-I. Castelli Risk Index-I.

## Data Availability

Raw data supporting the conclusions of this study will be made available by the authors upon request.
